# Editorial: Molecular mechanisms of Alzheimer’s disease: From top to bottom

**DOI:** 10.3389/fragi.2022.1026942

**Published:** 2022-09-16

**Authors:** Pâmela B. Mello-Carpes, Nibaldo C. Inestrosa, Andrea Cristina Paula-Lima

**Affiliations:** ^1^ Multicentric Physiological Sciences Graduate Program and Biochemistry Graduate Program, Federal University of Pampa, Uruguaiana, RS, Brazil; ^2^ Center for Aging and Regeneration, Department of Cell and Molecular Biology, Faculty of Biological Sciences, Pontifical Catholic University of Chile, Santiago, Chile; ^3^ Faculty of Medicine, Biomedical Neuroscience Institute, Universidad de Chile, Santiago, Chile; ^4^ Faculty of Dentistry, Institute for Research in Dental Sciences, Universidad de Chile, Santiago, Chile; ^5^ Department of Neurosciences, Faculty of Medicine, Universidad de Chile, Santiago, Chile

**Keywords:** tau pathology, amyloid–beta, oral-gut-brain axis, BDNF (brain derived neurotrophic factor), neurodegeneration

Alzheimer’s disease (AD) is a neurodegenerative disease related to aging. It is a type of senile dementia of high prevalence worldwide. It causes a gradual increase in irreversible cognitive decline that includes memory deficits, behavioral alterations and mental confusion, triggering devasting effects on daily life.

In AD, the damage and destruction of neurons gradually affect different parts of the brain. Neurodegeneration is preceded by the deposition of soluble oligomers and fibrils of the amyloid-β (Aβ) peptide in the extracellular space and by intracellular aggregation of the tau protein in neurofibrillary tangles (Pratico, 2008; Paula-Lima et al., 2013). According to the amyloid cascade, the accumulation of the extracellular Aβ peptide triggers the dysfunction in the release of neurotransmitters, extensive oxidative stress (Butterfield and Lauderback, 2002; Ill-Raga et al., 2010), synaptic failure and neuronal loss, leading to macroscopic atrophy (Lane et al., 2015).

Genetic and environmental factors are related to the risk of AD. Although current pharmacological treatments offer some relief from AD symptoms, the improvement is modest and temporary, indicating that the heterogeneity of the disease requires a stratified approach for effective treatment (Conway, 2020). Some non-pharmacological interventions have been investigated and show promissory effects. Between them, we highlighted the different types of physical exercise, which act by multiple neuroprotective mechanisms (Daré et al., 2020; Soares et al., 2021). However, until now, there is no curative treatment for AD, and researchers are still struggling to find therapeutic targets that promote significant improvements in the clinical conditions of Alzheimer’s patients (Knopman et al., 2021). In this context, comprehending the molecular mechanisms of this neurodegenerative pathology is critical to advances and developing effective interventions to break the pathology.

Research in AD is continuously evolving, and regularly new knowledge is provided. In this Research Topic, we present one mini-review of compelling evidence on the genetic connexion between AD and the mechanisms of regulation of the amyloid precursor protein (APP). Also, we present a review discussing additional risk factors for AD including chronic inflammatory diseases such as periodontitis, and their association with the gut functioning. Also, in a brief research report the authors described how epigenetic modifications triggered by inhibiting a neurotrophic pathway, are associated with oxidative stress *in vitro*. Finally, in an original research the authors described that the pattern of pTAU staining across the parietal-hippocampal network of the brain is a strong predictor of spatial learning and memory performance. Please find bellow the highlights of the work of this Research Topic:

1) Genetics and Amyloid-β pathology: In this mini-review article the authors focused on the mechanisms involved in the upregulation of Amyloid-β Precursor Protein (APP) expression. Sato et al. discussed the multiple transcriptional and post-transcriptional mechanisms of APP mRNA, and how alterations in these mechanisms could trigger AD development. The authors summarized the transcriptional enhancers and regulators of human APP. The understanding of human APP expression regulation could provide insights to AD treatment development.
2) Oral-gut-brain axis: Recently, the gut-brain axis has been implicated in the pathology (onset or progress) of many chronic diseases, which could be associated with an unbalanced oral-gut microbiota (dysbiosis). The oral microbiota is affected by oral diseases, such as Periodontitis, and the bacteria involved in this disease have been detected in distal tissues, indicating an oral-gut communication. Of particular interest, periodontitis has been increasingly pointed out as a risk factor for AD. In an interesting review, Sansores-España et al. explore the bi-directional relationship of the gut-brain and oral-brain axis and discuss how dementia could have its onset in oral or intestinal dysbiosis.
3) Epigenetics: Histone modifications are related to neurodegenerative diseases such as AD. Evidence suggests that histone 3, lysine 9 trimethylated (H3K9me3) is related to aging and related diseases, including cognitive decline, regulating brain-derived neurotrophic factor (BDNF), neuron survival, and brain plasticity. In a brief report, Ionescu-Tucker et al. addressed the role of age-associated stressors on H3K9me3 regulation. Using cultured hippocampal neurons, they demonstrated that inhibition of BDNF signaling elevates hippocampal H3K9me3 in a manner dependent on *in vitro* age and oxidative stress. These data support the importance of epigenetics regulation of hippocampal BDNF, a neurotrophin that has a key role in learning and memory.
4) Tau and amyloid-β pathology: Despite spatial navigational impairments being early events in AD, the specific brain changes associated with spatial learning and memory deficits are not entirely understood. In an original article, Stimmell et al., using a triple transgenic mouse model for AD (3xTg-AD) mice that express plaques and tangles, examined the relationship between Tau pathology profile across the parietal-hippocampal brain network, and its association with spatial learning and memory. The authors conclude that the pattern of pTau staining across the parietal-hippocampal network is a strong predictor of spatial learning and memory performance.

The articles composing this Research Topic provide evidence that contributes to a better understanding of AD mechanisms. They highlighted new aspects of AD pathology and reviewed what is already known about critical open issues. The set of data delineated here helps the understanding of AD on different but related aspects, sharing potential starting points, especially for developing treatment strategies.
